# Subaortic and midventricular obstructive hypertrophic cardiomyopathy with extreme segmental hypertrophy

**DOI:** 10.1186/1476-7120-5-12

**Published:** 2007-03-12

**Authors:** Georgios K Efthimiadis, Georgios Giannakoulas, Despina G Parcharidou, Antonios G Ziakas, Christodoulos E Papadopoulos, Takis Karoulas, Christodoulos Pliakos, Georgios Parcharidis

**Affiliations:** 1 Cardiology Department, AHEPA Hospital, Aristotle University of Thessaloniki, Thessaloniki, Greece

## Abstract

**Background:**

Subaortic and midventricular hypertrophic cardiomyopathy in a patient with extreme segmental hypertrophy exceeding the usual maximum wall thickness reported in the literature is a rare phenomenon.

**Case Presentation:**

A 19-year-old man with recently diagnosed hypertrophic cardiomyopathy (HCM) was referred for sudden death risk assessment. The patient had mild exertional dyspnea (New York Heart Association functional class II), but without syncope or chest pain. There was no family history of HCM or sudden death. A two dimensional echocardiogram revealed an asymmetric type of LV hypertrophy; anterior ventricular septum = 49 mm; posterior ventricular septum = 20 mm; anterolateral free wall = 12 mm; and posterior free wall = 6 mm. The patient had 2 types of obstruction; a LV outflow obstruction due to systolic anterior motion of both mitral leaflets (Doppler-estimated 38 mm Hg gradient at rest); and a midventricular obstruction (Doppler-estimated 43 mm Hg gradient), but without apical aneurysm or dyskinesia. The patient had a normal blood pressure response on exercise test and no episodes of non-sustained ventricular tachycardia in 24-h ECG recording. Cardiac MRI showed a gross late enhancement at the hypertrophied septum. Based on the extreme degree of LV hypertrophy and the myocardial hyperenhancement, an implantation of a cardioverter-defibrillator was recommended prophylactically for primary prevention of sudden death.

**Conclusion:**

Midventricular HCM is an infrequent phenotype, but may be associated with an apical aneurysm and progression to systolic dysfunction (end-stage HCM).

## Background

Midventricular hypertrophic cardiomyopathy (HCM) is an infrequent phenotype, but may be associated with an apical aneurysm and progression to systolic dysfunction (end-stage HCM) [1].

## Case presentation

**Figure 1:**
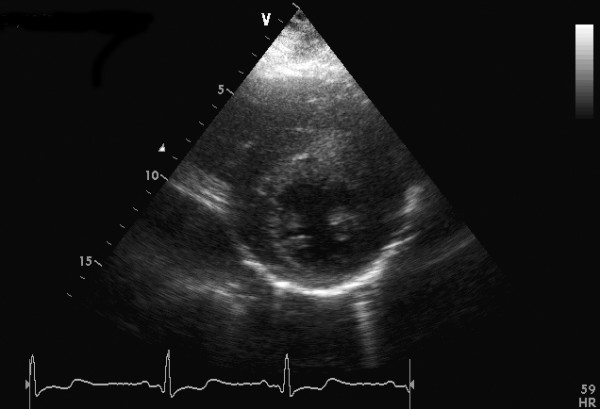
Short-axis view at end diastole showing an asymmetric type hypertrophic cardiomyopathy with extreme hypertrophy of the anterior septum.

**Figure 2:**
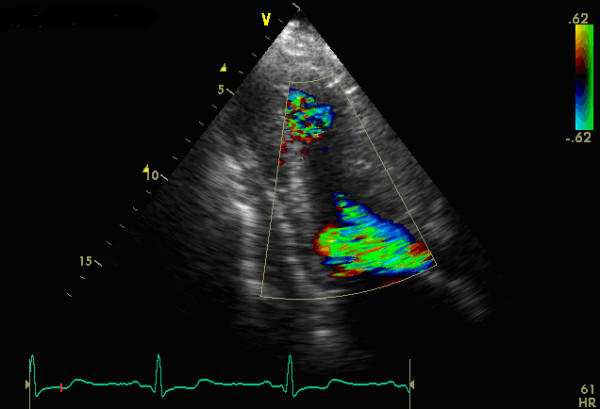
A three-chamber view showing a combination of 2 different flow jets, one in the mid of left ventricle and the other in the outflow tract.

A 19-year-old man with recently diagnosed HCM was referred for sudden death risk assessment. The patient had mild exertional dyspnea (New York Heart Association functional class II), but without syncope or chest pain. There was no family history of HCM or sudden death. The patient's blood pressure was 130/70 mmHg and a grade 3–4/6 systolic ejection murmur was present at the apex. ECG showed a left ventricular (LV) hypertrophy with strain pattern. A two dimensional echocardiogram revealed an asymmetric type of LV hypertrophy; anterior ventricular septum = 49 mm; posterior ventricular septum = 20 mm; anterolateral free wall = 12 mm; and posterior free wall = 6 mm (Figure 1). The patient had 2 types of obstruction; a LV outflow obstruction due to systolic anterior motion of both mitral leaflets (Doppler-estimated 38 mm Hg gradient at rest); and a midventricular obstruction (Doppler-estimated 43 mm Hg gradient), but without apical aneurysm or dyskinesia (Figure 2) [see Additional file 1]. The LV end diastolic diameter was 45 mm and the left atrium was 45 mm. The patient had a normal blood pressure response on exercise test and no episodes of non-sustained ventricular tachycardia in 24-h ECG recording. Cardiac MRI showed a gross late enhancement at the hypertrophied septum. Based on the extreme degree of LV hypertrophy and the myocardial hyperenhancement, an implantation of a cardioverter-defibrillator was recommended prophylactically for primary prevention of sudden death. Written consent was obtained from the patient for publication of study.

This case is a rare example of a patient with subaortic and midventricular hypertrophic cardiomyopathy with extreme segmental hypertrophy exceeding the usual maximum wall thickness reported in the literature, although Maron et al have published a case of a patient with an even greater hypertrophy (60 mm) [2].

## Supplementary Material

Additional File 1 Movie representing 2 types of obstruction. This movie shows a three-chamber view with a combination of 2 different flow jets, one in the mid of left ventricle and the other in the outflow tract. Click here for file
